# Diabetes concomitant to aortic stenosis is associated with increased expression of NF-κB and more pronounced valve calcification

**DOI:** 10.1007/s00125-021-05545-w

**Published:** 2021-09-07

**Authors:** Magdalena Kopytek, Piotr Mazur, Michał Ząbczyk, Anetta Undas, Joanna Natorska

**Affiliations:** 1 John Paul II Hospital, Kraków, Poland; 2 Jagiellonian University Medical College, Kraków, Poland

**Keywords:** Aortic stenosis, Bone morphogenetic protein 2, Coagulation factors, Diabetes mellitus, Inflammation, NF-κB, Oxidative stress

## Abstract

**Aims/hypothesis:**

Type 2 diabetes has been demonstrated to predispose to aortic valve calcification. We investigated whether type 2 diabetes concomitant to aortic stenosis (AS) enhances valvular inflammation and coagulation activation via upregulated expression of NF-κB, with subsequent increased expression of bone morphogenetic protein 2 (BMP-2).

**Methods:**

In this case–control study, 50 individuals with severe isolated AS and concomitant type 2 diabetes were compared with a control group of 100 individuals without diabetes. The median (IQR) duration of diabetes since diagnosis was 11 (7–18) years, and 36 (72%) individuals had HbA_1c_ ≥48 mmol/mol (≥6.5%). Stenotic aortic valves obtained during valve replacement surgery served for in loco NF-κB, BMP-2, prothrombin (FII) and active factor X (FXa) immunostaining. In vitro cultures of valve interstitial cells (VICs), isolated from obtained valves were used for mechanistic experiments and PCR investigations.

**Results:**

Diabetic compared with non-diabetic individuals displayed enhanced valvular expression of NF-κB, BMP-2, FII and FXa (all *p* ≤ 0.001). Moreover, the expression of NF-κB and BMP-2 positively correlated with amounts of valvular FII and FXa. Only in diabetic participants, valvular NF-κB expression was strongly associated with serum levels of HbA_1c_, and moderately with fructosamine. Of importance, in diabetic participants, valvular expression of NF-κB correlated with aortic valve area (AVA) and maximal transvalvular pressure gradient. In vitro experiments conducted using VIC cultures revealed that glucose (11 mmol/l) upregulated expression of both NF-κB and BMP-2 (*p* < 0.001). In VIC cultures treated with glucose in combination with reactive oxygen species (ROS) inhibitor (*N*-acetyl-l-cysteine), the expression of NF-κB and BMP-2 was significantly suppressed. A comparable effect was observed for VICs cultured with glucose in combination with NF-κB inhibitor (BAY 11–7082), suggesting that high doses of glucose activate oxidative stress leading to proinflammatory actions in VICs. Analysis of mRNA expression in VICs confirmed these findings; glucose caused a 6.9-fold increase in expression of *RELA* (NF-κB p65 subunit), with the ROS and NF-κB inhibitor reducing the raised expression of *RELA* by 1.8- and 3.2-fold, respectively.

**Conclusions/interpretation:**

Type 2 diabetes enhances in loco inflammation and coagulation activation within stenotic valve leaflets. Increased valvular expression of NF-κB in diabetic individuals is associated not only with serum HbA_1c_ and fructosamine levels but also with AVA and transvalvular gradient, indicating that strict long-term glycaemic control is needed in AS patients with concomitant type 2 diabetes. This study suggests that maintaining these variables within the normal range may slow the rate of AS progression.

**Graphical abstract:**

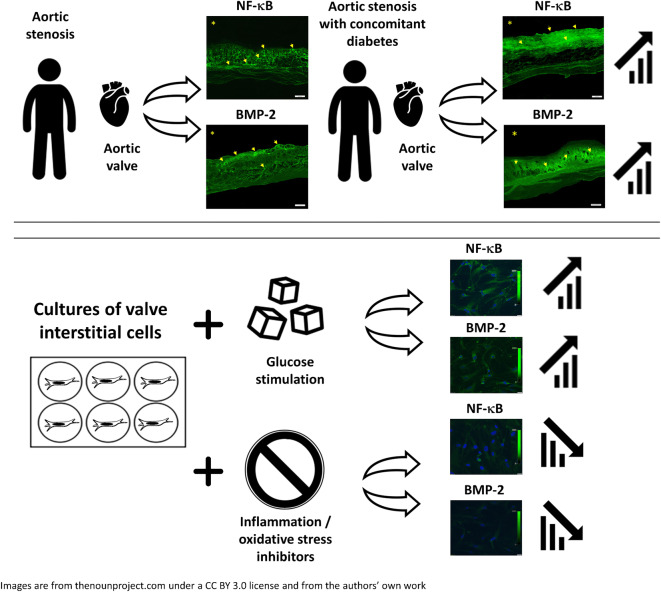

**Supplementary Information:**

The online version contains peer-reviewed but unedited supplementary material available at 10.1007/s00125-021-05545-w.

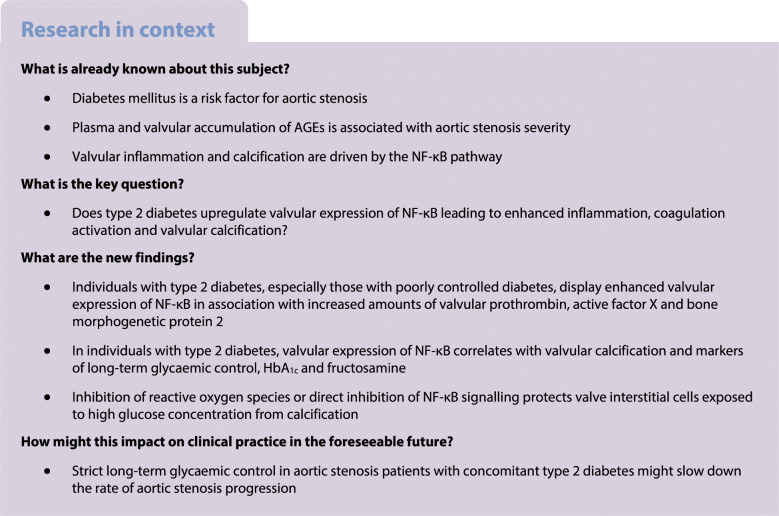



## Introduction

Aortic stenosis (AS) is a progressive disease associated with reduction of the aortic valve orifice and leaflet mobility due to a build-up of calcium. A consequence of this defect is an impaired blood ejection from the left ventricle into the aorta. AS is the most common acquired valvular heart disease in the western adult population, with no available pharmacological treatment. The prevalence of AS in individuals >65 years of age ranges between 2% and 7% [1]. It is estimated that 4.5 million cases of AS will be present worldwide by the year 2030 [2]. Aortic valve replacement, whether surgical or percutaneous, is the only definitive treatment for AS. While both methods present excellent outcomes, surgical intervention remains the treatment of choice for the vast majority of patients [3].

The initial stage of aortic valve degeneration is endothelial damage by high shear stress [4–6]. Then, subendothelial accumulation of intracellular lipids, lipoproteins and mediators of calcification is observed, together with activation of local and systemic inflammation [7, 8].

AS shares some risk factors with atherosclerosis. Among people with AS, similar to atherosclerosis, the prevalence of diabetes is visibly higher than in the general population and appears to be increasing [9, 10]. Ljungberg et al [11] have shown in population-based cohorts in northern Sweden that the prevalence of diabetes 10 years before surgery for AS was 15.8%.

Hyperglycaemia has been proposed as one of the metabolic states enhancing aortic valve fibrosis and calcification [12–14] through a complex mechanism involving increased valvular protein glycation, of reactive oxygen species (ROS) generation, inflammation and coagulation activation [15, 16]. Although the pivotal mechanism leading to such dysregulation is not fully understood, formation of AGEs has been suggested as a factor initiating and/or escalating valvular calcification [17, 18].

Our previous study showed increased valvular expression of C-reactive protein (CRP) and its mRNA, and higher tissue factor (TF) expression in individuals with AS and concomitant type 2 diabetes compared with non-diabetic individuals [14]. Moreover, regulation of valvular inflammation is under control of NF-κB [19]. In loco activation of NF-κB leads to an upregulation of IL-6, implicated in calcification of aortic valves via bone morphogenetic protein (BMP) stimulation [20]. In addition, coagulation factors such as TF and active factor X (FXa) upregulate inflammation and fibrosis through NF-κB signalling [21, 22].

Here, we hypothesised that metabolic dysregulation seen in type 2 diabetes may lead to enhanced valvular NF-κB expression. Thus, we investigated the valvular expression of NF-κB, BMP-2 and components of the blood coagulation system in individuals with AS and concomitant type 2 diabetes.

## Methods

### Participants

Between August 2016 and April 2019, we recruited 50 individuals with isolated symptomatic AS and concomitant type 2 diabetes and a control group of 100 individuals with AS without diabetes of similar age and sex. All participants underwent first-time elective surgical aortic valve replacement at the Department of Cardiovascular Surgery and Transplantology at the John Paul II Hospital, Krakow, Poland [18]. Data on demographics, medical history and current treatment were collected using a standardised questionnaire. AS was diagnosed based on transthoracic echocardiography performed by an experienced cardiologist on a Toshiba APLIO 80 (Toshiba, Tokyo, Japan) ultrasound machine, and it was defined as a mean transvalvular pressure gradient (PG_mean_) ≥40 mmHg and/or aortic valve area (AVA) <1 cm^2^ [23]. Arterial hypertension was diagnosed based on a history of hypertension (BP >140/90 mmHg) or preadmission antihypertensive treatment. Type 2 diabetes was diagnosed based on fasting serum glucose ≥7.0 mmol/l on two separate occasions, HbA_1c_ ≥48 mmol/mol (6.5%), or post-load plasma glucose levels ≥11.1 mmol/l [24]. All participants had diabetes diagnosed at least 5 years before enrolment and all were receiving treatment with insulin or oral glucose-lowering agents. To exclude latent autoimmune diabetes in adults (LADA), GAD65 antibodies and C-peptide concentrations were assessed in the diabetic participants. Participants negative for GAD autoantibodies and who had C-peptide within the normal range were classified as having type 2 diabetes. Twenty-four hours prior to aortic valve replacement, all participants receiving oral glucose-lowering agents were switched to insulin. Fasting blood glucose and HbA_1c_ levels were routinely performed in all diabetic participants and in the non-diabetic participants with AS who served as a control group.

Hypercholesterolaemia was diagnosed based on medical records, cholesterol-lowering therapy, or total cholesterol ≥5.0 mmol/l. Smoking was defined as the use of one or more cigarettes per day.

The following exclusion criteria were applied: atherosclerosis requiring concomitant revascularisation; rheumatic AS; acute infection including infective endocarditis; diagnosed malignancy; chronic kidney disease; previous pericardiotomy; required concomitant valvular surgery (e.g. mitral valve repair); recent (<90 days) acute coronary syndrome or cerebrovascular episode; percutaneous coronary intervention; and pregnancy. The valvular anatomy was identified preoperatively by echocardiography and confirmed intraoperatively by a cardiac surgeon. Bicuspid valve and root/ascending aortic dilatation requiring intervention were used as an exclusion criterion. The diagnosis of atherosclerosis was based on angiographically documented coronary artery stenosis >20% diameter and such individuals were excluded from the study.

The Ethical Committee (Krakow District Medical Chamber, Poland) approved the study and all participants provided written informed consent in accordance with the Declaration of Helsinki*.*

### Laboratory analysis

At 07:00–09:00 hour, before surgical aortic valve replacement, fasting venous blood was drawn from the antecubital veins. Citrated blood was centrifuged at 2500 *g* at 20°C for 20 min, while blood drawn into serum or EDTA tubes was centrifuged at 1600 *g* at 4°C for 10 min. All samples were stored in small aliquots at −80°C until analysis. Routine laboratory assays were used to determine lipid profile, glucose, creatinine, CRP and fibrinogen. HbA_1c_ was assessed using a turbidimetric inhibition immunoassay (TINIA) in whole-blood haemolysates (Roche Diagnostics, Mannheim, Germany). Serum fructosamine levels were measured using a colorimetric assay (Roche Diagnostics, Risch-Rotkreuz, Switzerland).

### Aortic valve preparation

Valves were collected during open heart surgeries and transferred directly from the operating room to the laboratory. One valvular leaflet was used for in loco analysis and two for in vitro studies (cell cultures and mRNA expression). Valve leaflets were cryosectioned into 4.5 μm sections as previously described [14, 18, 25].

### Immunofluorescence analysis

Immunostaining was performed on 50 valves obtained from diabetic individuals and on 50 randomly selected valves from individuals with AS without diabetes, according to the previously described protocol [14, 18]. Specific primary antibodies were used against NF-κB (p65 subunit, 1:500), BMP-2 (1:200), prothrombin (FII, 1:100) and FXa (1:200) (all from Abcam, Cambridge, UK) by overnight incubation at 4°C. The corresponding secondary goat or mouse antibodies conjugated with AlexaFluor 488 (Abcam) (1:1000) were applied in the dark at 4°C for 1 h. A negative control, without primary antibody was performed for all staining. All analyses were repeated three times. The Olympus BX 43 microscope (Tokyo, Japan) equipped with dedicated software (cellSense Dimension 2.3, License Version 2, Serial Number: BRR7BPW2NQP; Münster, Germany) was used to analyse images. Positively stained areas were assessed on a continuum from the undetected level (0%) to diffused staining (100%) and were calculated by two independent observers from 30 images taken of each valve. The percentages of immunopositive areas were calculated as the extent of positive immunoreactive areas/total sample area [25]. The fluorescence intensity was computed as the ratio (%) of positively and negatively stained areas. The investigators were blinded to the sample origin. The intra-observer variability was below 6%.

### ELISAs

Active factor VIIa–antithrombin complex (FVIIa-AT; Diagnostica Stago, Asnières-sur-Seine, France), TF (R&D System, Minneapolis, MN, USA) and prothrombin fragments 1+2 (F1+2; Siemens Healthcare, Marburg, Germany) were assayed quantitatively in plasma samples using commercial ELISAs in accordance with manufacturers’ instructions.

### Valve interstitial cells in vitro cultures

Valve interstitial cells (VICs) were isolated and cultured as previously described [26]. All experiments were performed on VICs between their third and fifth passages. To initiate calcification, VICs were cultured in a calcification medium containing β-glycerophosphate disodium hydrate salt (10 mmol/l; Sigma-Aldrich, St Louis, MO, USA), CaCl_2_ (1.5 mmol/l; Chempur, Piekary Slaskie, Poland) and ascorbic acid (50 μg/ml; Chempur) and stimulated or not (a negative control) with TNF-α (50 ng/ml). In parallel, to investigate the influence of glucose, VICs were cultured in the calcification medium supplemented with the d-(+)-glucose (11 mmol/l; Sigma-Aldrich). BMP-2 was used as a marker of calcification and was quantified using immunofluorescence as described above. To inhibit oxidative stress generated by high concentration of glucose, the inhibitor of ROS was added (1 mmol/l *N*-acetyl-l-cysteine [NAC]; Sigma-Aldrich) to the calcification medium 1 h before glucose stimulation. Similarly, to inhibit the transcription pathway of NF-κB, an inhibitor (BAY 11-7082; Sigma-Aldrich) was added to the calcification medium at a concentration of 10^−6^ mol/l 30 min before glucose stimulation. VICs were cultured for 72 h. Each experiment was repeated three times using VICs isolated from another valve.

### Relative quantification of transcripts by real-time PCR

A total of 400 ng of RNA from VICs was reverse transcribed to single-strand cDNA (Applied Biosystems, Foster City, CA, USA) according to the manufacturer’s instruction. The cDNA was amplified with TaqMan Gene Expression Assay (Hs01042014_m1 for NF-κB p65 Rel; gene symbol: *RELA*) containing both primers and probe on an ABI PRISMR 7900HT Fast Real-Time PCR System (Applied Biosystems). β-Actin (Hs99999903_m1, human ACTB Endogenous Control FAM/ MGB Probe, Non-Primer Limited; Applied Biosystems) was used as a housekeeping gene. To analyse the obtained data, the comparative threshold cycle method was applied [26].

### Statistical analyses

All statistical analyses were performed using STATISTICA Version 13.3 (TIBCO Software, Palo Alto, CA, USA) software. Categorical variables were presented as numbers and percentages and were analysed by Pearson’s χ^2^ or two-tailed Fisher’s exact test. Continuous variables were expressed as mean ± SD or median (IQR). Normality was analysed by the Shapiro–Wilk test. Differences between groups were compared using Student’s *t* test for normally distributed variables or the Mann–Whitney *U* test for non-normally distributed variables. Associations between normally distributed continuous variables were calculated using Pearson’s correlation coefficient, while non-parametric variables were assessed by Spearman’s test. A *p* value of <0.05 was considered statistically significant.

## Results

Baseline characteristics of participants with AS, with and without type 2 diabetes, are shown in Table 1. The median duration of diabetes was 11 (7–18) years and 36 (72%) individuals had HbA_1c_ ≥48 mmol/mol (≥6.5%).

**Table 1:** Baseline characteristics of participants with AS, with or without concomitant type 2 diabetes

Variable	Diabetic participants (*n*=50)	Non-diabetic participants (*n*=100)	*p* value^a^
Age, years	70.2 ± 6.2	67.8 ± 5.6	0.08
Female sex, *n* (%)	31 (62)	55 (55)	0.41
BMI, kg/m^2^	31.3 (28.7–34.5)	28.3 (26.6–30.9)	0.049
Risk factors, *n* (%)			
Arterial hypertension	50 (100)	90 (90)	0.05
Hypercholesterolaemia	46 (92)	84 (84)	0.21
Current smoking	8 (16)	18 (18)	0.76
Medications, *n* (%)			
β-Blockers	47 (94)	87 (87)	0.19
Aspirin	40 (80)	76 (76)	0.58
ACE inhibitors	45 (90)	85 (85)	0.40
Statins	46 (92)	76 (76)	0.025
Insulin	14 (28)	0	<0.0001
Metformin	36 (72)	0	<0.0001
Echocardiographic data			
Mean gradient, mmHg	52 (43–66)	47 (43–58)	0.047
Maximal gradient, mmHg	89.2 ± 12.3	80 ± 14.2	0.042
LVEF, %	60 (58–64)	59 (50–65)	0.22
AVA, cm^2^	0.78 (0.60–0.82)	0.87 (0.72–0.91)	0.044
Laboratory investigation			
Fibrinogen, g/l	3.6 ± 0.6	3.3 ± 0.76	0.3
Creatinine, μmol/l	81 (74–100)	82 (65–95)	0.68
CRP, mg/l	1.0 (1.0–2.0)	1.8 (1.0–4.0)	0.29
Glucose, mmol/l	7.5 (5.9–9.1)	5.3 (5.0–5.6)	<0.0001
HbA_1c_, mmol/mol	51 (45–62)	37 (34–40)	<0.0001
HbA_1c_, %	6.8 (6.3–7.8)	5.5 (5.3–5.8)	<0.0001
Fructosamine, μmol/l	262 (241–291)	223 (220–239)	0.007
TC, mmol/l	3.8 (3.0–4.6)	4.0 (3.6–4.7)	0.11
LDL-cholesterol, mmol/l	2.3 (1.5–3.1)	2.5 (2.0–3.4)	0.12
HDL-cholesterol, mmol/l	1.2 (1.0–1.4)	1.5 (1.2–1.5)	0.12
Triacylglycerols, mmol/l	1.5 (1.2–2.0)	1.4 (1.0–1.9)	0.39

Data are presented as *n* (%), mean±SD or median (IQR)

^a^Categorical variables were analysed by the χ^2^ test; the Mann–Whitney *U* or Student’s *t* tests were used to compare differences between groups

ACE, angiotensin converting enzyme; DM, type 2 diabetes; LVEF, left ventricular ejection fraction; TC, total cholesterol

In the whole population of diabetic participants, no associations were found between serum glucose, HbA_1c_ or fructosamine levels and echocardiographic variables. However, diabetic participants with HbA_1c_ ≥48 mmol/mol (≥6.5%) compared with HbA_1c_ <48 mmol/mol (<6.5%) were characterised by 32% higher maximal transvalvular pressure gradient (PG_max_; 87 [64–99] vs 66 [63–80] mmHg, *p* = 0.038), 18% higher PG_mean_ (52 [43–65] vs 44 [42–51] mmHg, *p* = 0.036) and 18% lower AVA (0.7 [0.6–0.8] vs 0.85 [0.8–0.9] cm^2^, *p* = 0.0005).

### In loco studies

#### Valvular expression of NF-κB in association with valve calcification

**Fig. 1:**
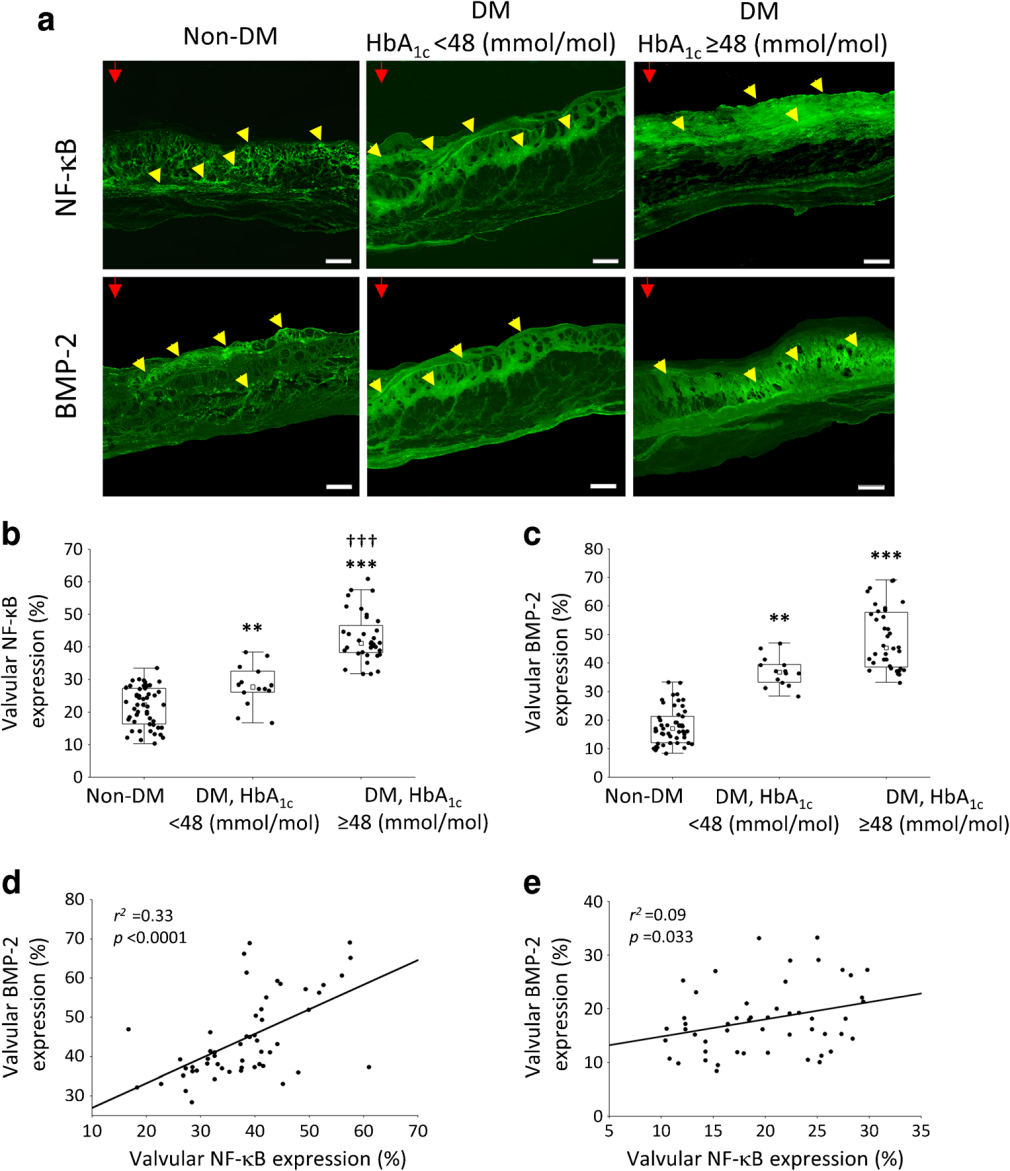
The expression of NF-κB and BMP-2 within stenotic aortic valves in participants with AS and concomitant type 2 diabetes compared with participants with AS without diabetes. (**a**) Representative microphotographs of valvular NF-κB and BMP-2 expression (red arrowheads indicate aortic side of the leaflet; yellow arrowheads indicate the immunopositive area of expression). Scale bar, 200 μm. (**b**, **c**) Box plots showing valvular expression of NF-κB (**b**) and BMP-2 (**c**). Values are medians (IQR). **p<0.01 and ***p<0.001 vs non-DM; ^†††^*p*<0.001 vs DM with HbA_1c_ <48 mmol/mol (<6.5%). (**d**, **e**) Associations between valvular expression of NF-κB and BMP-2 in participants with AS with (**d**) and without (**e**) concomitant diabetes. DM, AS with concomitant type 2 diabetes; Non-DM, AS without concomitant diabetes

**Fig. 2:**
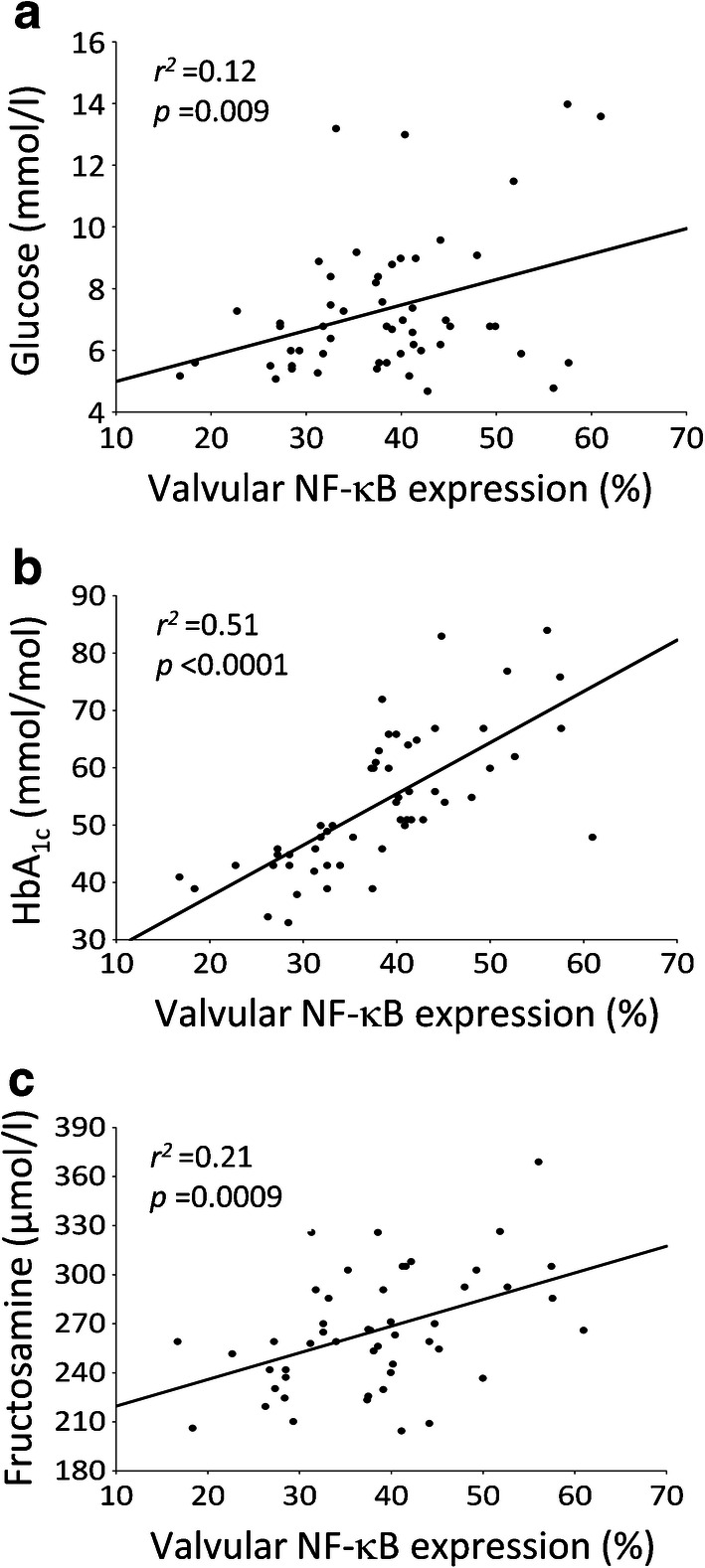
Associations between valvular NF-κB expression and serum markers of glycaemic control in participants with AS and concomitant type 2 diabetes. Scatterplots represent the correlation between valvular NF-κB expression and serum levels of glucose (**a**), valvular NF-κB expression and serum concentrations of HbA_1c_ (**b**), and valvular NF-κB expression and serum levels of fructosamine (**c**)

NF-κB valvular expression was observed mainly on the aortic side of the leaflets, in both diabetic and control participants (Fig. 1). However, valves from diabetic compared with control participants were characterised by a 92% higher level of NF-κB expression (38 ± 10% vs 20 ± 6%, *p* = 0.001). In non-diabetic participants, expression of NF-κB presented a diffused pattern of fluorescence, while within valves from diabetic participants the expression was more condensed (Fig. 1a). Interestingly, the highest expression of NF-κB was found in the diabetic participants with HbA_1c_ ≥48 mmol/mol (≥6.5%) (+45%) (Fig. 1b). A similar pattern of immunofluorescence was observed with regard to valvular calcification, reflected by 148% (*p* < 0.001) higher BMP-2 expression in diabetic participants compared with control participants (Fig. 1a), with the highest percentage of BMP-2-positive areas (+23%) in diabetic participants with HbA_1c_ ≥48 mmol/mol (≥6.5%) compared with those with HbA_1c_ <48 mmol/mol (<6.5%) (Fig. 1c). Moreover, positive associations between valvular NF-κB and BMP-2 were found in both diabetic and non-diabetic participants (Fig. 1d,e). Only in the diabetic group valvular NF-κB expression was weakly associated with serum glucose (Fig. 2a), strongly associated with HbA_1c_ (Fig. 2b) and moderately with fructosamine (Fig. 2c). In diabetic participants, valvular expression of BMP-2 correlated with HbA_1c_ (*r*^*2*^ = 0.65, *p* < 0.0001) and fructosamine levels (*r*^*2*^ = 0.15, *p* = 0.006) but not with glucose. No such associations were noted for control participants with AS but without concomitant diabetes.

#### Valvular expression of coagulation factors in association with NF-κB and BMP-2

**Fig. 3:**
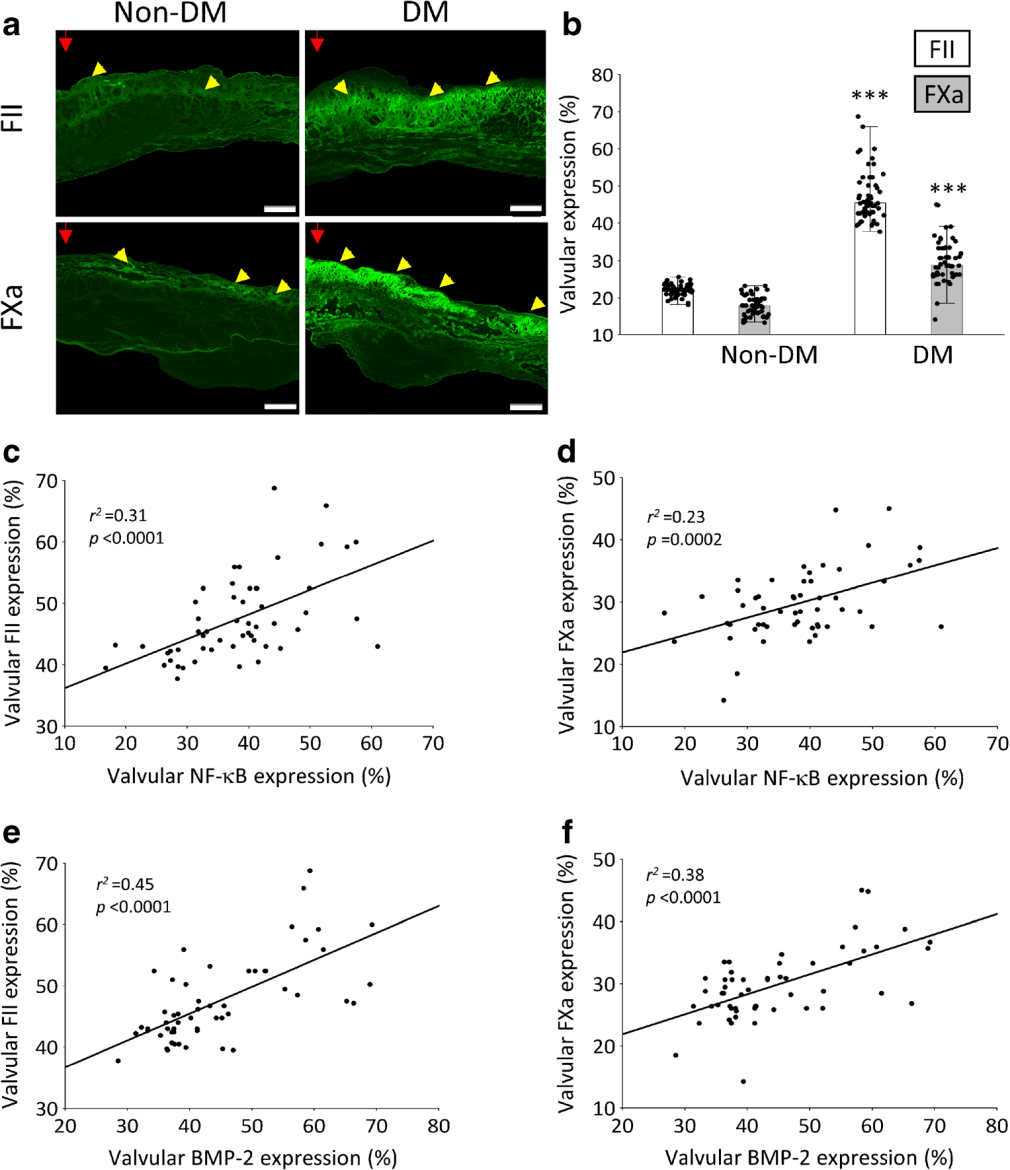
The expression of valvular FII and FXa within stenotic aortic valves in participants with AS and concomitant type 2 diabetes compared with participants with AS without diabetes. (**a**) Representative microphotographs of valvular FII and FXa expression (red arrowheads indicate aortic side of the leaflet; yellow arrowheads indicate the immunopositive area of expression). Scale bar, 200 μm. (**b**) Bar graph showing valvular expression levels of FII and FXa. Values are medians (IQR).***p<0.001 vs non-DM. (**c**–**f**) The scatterplots show correlations between valvular NF-κB and FII (**c**), NF-κB and FXa (**d**), BMP-2 and FII (**e**), and BMP-2 and FXa (**f**) in participants with AS and concomitant type 2 diabetes. DM, AS with concomitant type 2 diabetes; Non-DM, AS without concomitant diabetes

In control participants with AS but without concomitant diabetes the valvular expression of FII and FXa was detected on the aortic side of the leaflets, in the endothelial and subendothelial layers, while in participants with type 2 diabetes the expression of both proteins was observed additionally in the fibrosa layer (Fig. 3a). Compared with valves from control participants, valves from diabetic patients were characterised by 113% higher expression levels of FII and 66% higher expression levels of FXa (both *p* < 0.001) (Fig. 3b). The expression of both factors was slightly higher (both *p* > 0.05) in diabetic participants with HbA_1c_ ≥48 mmol/mol (≥6.5%), compared with those with HbA_1c_ <48 mmol/mol (<6.5%). In diabetic participants, valvular NF-κB correlated positively with FII and FXa expression (Fig. 3c,d). Similar associations were observed in participants without diabetes (electronic supplementary material [ESM] Fig. 1a,b). Moreover, in diabetic participants, valvular BMP-2 was positively associated with the expression of FII and FXa (Fig. 3e,f). Both factors were co-expressed with BMP-2. In control participants, valvular FXa (*r*^2^ = 0.13, *p* = 0.01) but not FII (*p* = 0.38) correlated positively with valvular BMP-2.

#### Associations of valvular factors with echocardiographic variables

**Fig. 4:**
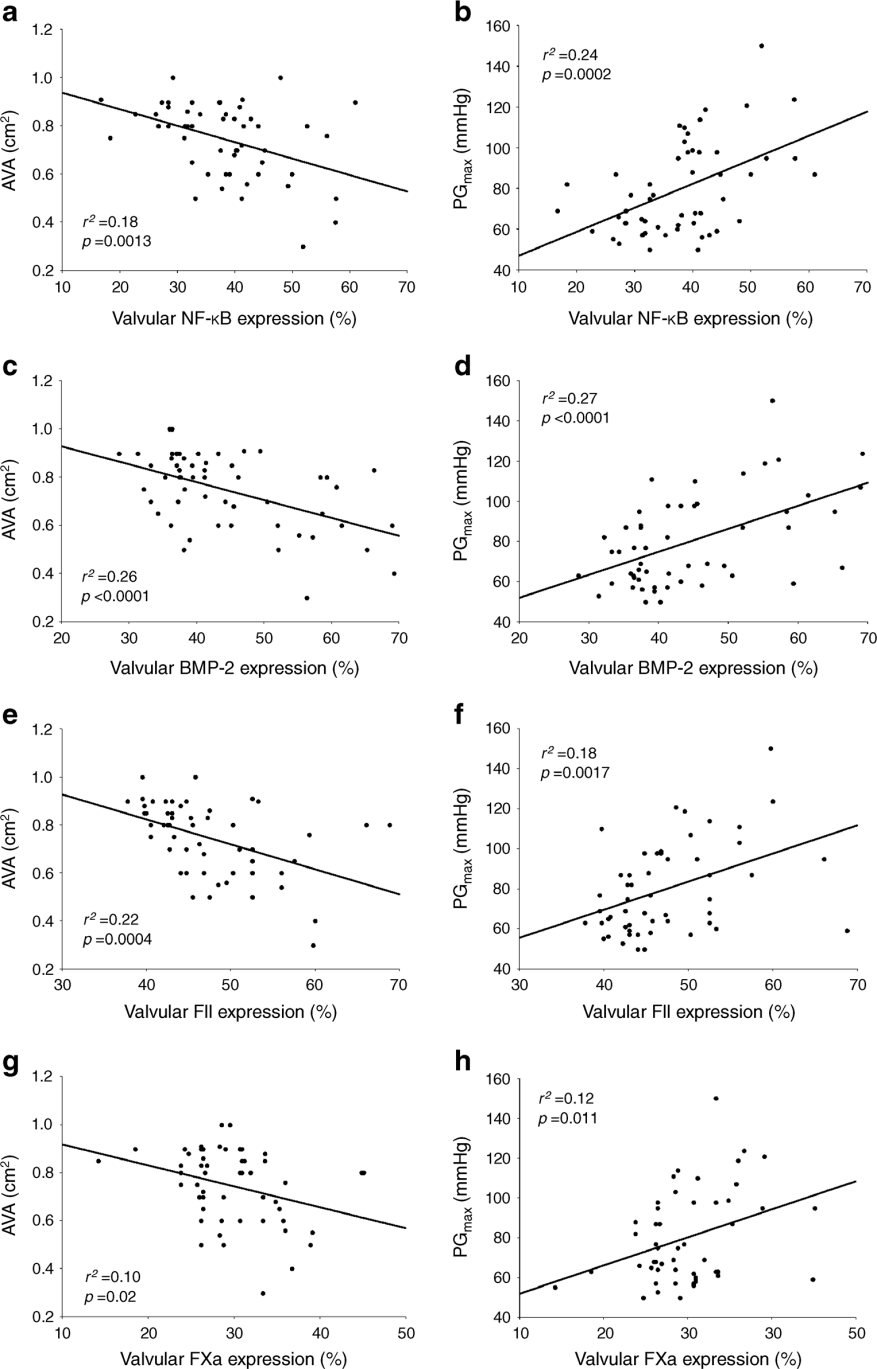
Associations between valvular expression of inflammatory, calcification and coagulation factors and disease severity in participants with AS and concomitant type 2 diabetes. The scatterplots show correlations between valvular NF-κB and AVA (**a**), valvular NF-κB and PG_max_ (**b**), valvular BMP-2 and AVA (**c**), valvular BMP-2 and PG_max_ (**d**), valvular FII and AVA (**e**), valvular FII and PG_max_ (**f**), valvular FXa and AVA (**g**), and valvular FXa and PG_max_ (**h**)

In participants with AS and concomitant type 2 diabetes, valvular NF-κB expression correlated with AVA and PG_max_ (Fig. 4a,b). In the control group of participants, we found the inverse association solely between valvular NF-κB and AVA (ESM Fig. 1c). In diabetic participants, we also observed that valvular BMP-2 expression was associated with AVA and PG_max_ (Fig. 4c,d), while in control participants, BMP-2 expression correlated solely with AVA (ESM Fig. 1d). In diabetic participants both valvular FII and FXa were associated with AVA (Fig. 4e,g) and PG_max_ (Fig. 4f,h). Even when participants were matched based on PG_max_ (median [IQR] 87 [75–95] for diabetic participants vs 90 [83–94] mmHg for control participants, *p* = 0.36), those with type 2 diabetes (*n* = 17) vs without diabetes (*n* = 19) had higher valvular expression levels of NF-κB (+77%, *p* < 0.0001), BMP-2 (+118%, *p* < 0.0001), FII (+107%, *p* < 0.0001) and FXa (+65%, *p* < 0.0001).

#### Plasma markers of coagulation

**Fig. 5:**
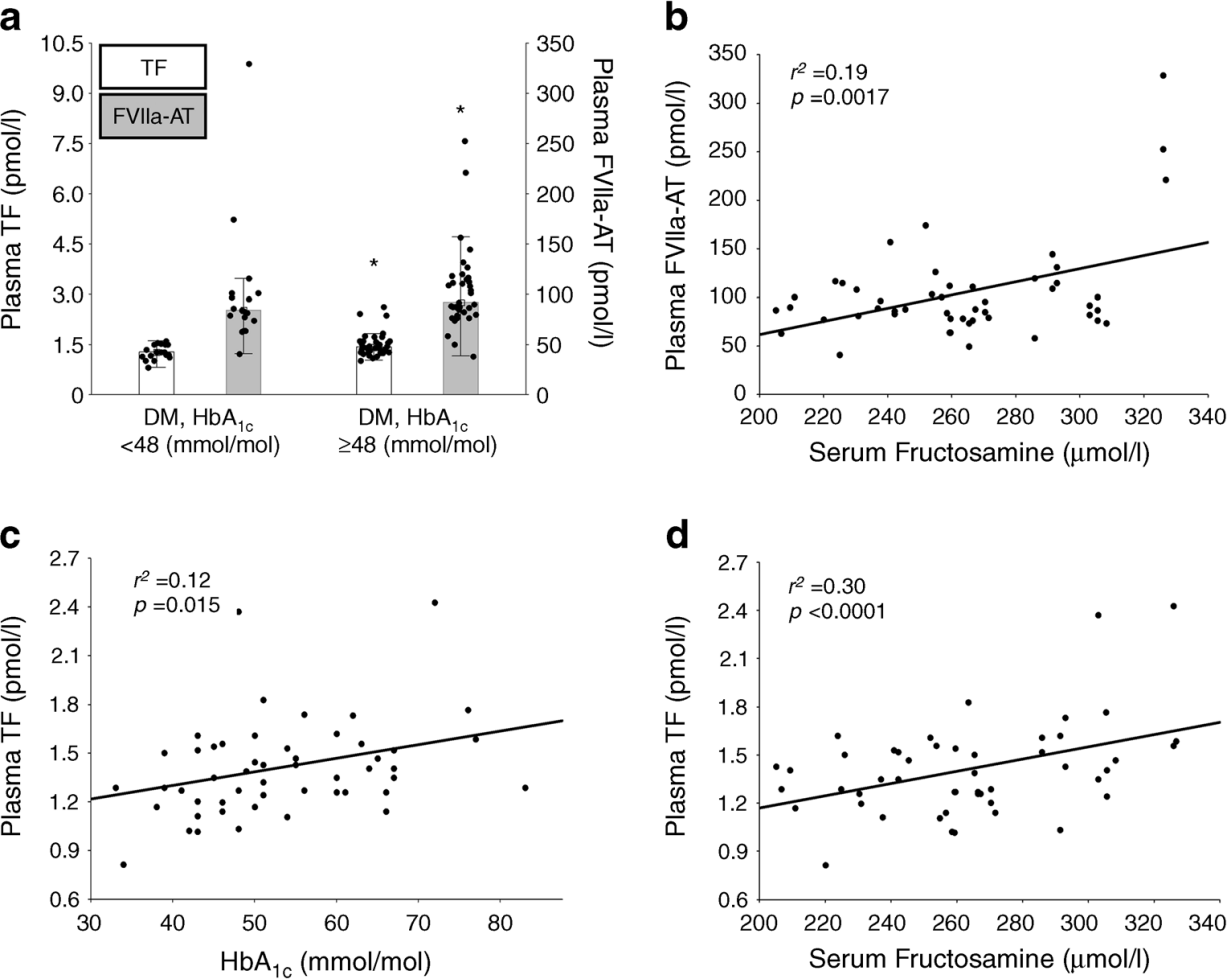
Plasma levels of TF and FVIIa-AT in participants with AS and concomitant type 2 diabetes. (**a**) Bar graphs showing plasma levels of TF and FVIIa-AT in diabetic participants with HbA_1c_ <48 mmol/mol (<6.5%) and HbA_1c_ ≥48 mmol/mol (≥6.5%). Values are medians (IQR). *p<0.05 vs DM with HbA_1c_ <48 (mmol/mol). (**b**–**d**) The scatterplots show correlations between serum levels of fructosamine and plasma concentrations of FVIIa-AT (**b**), serum concentrations of HbA_1c_ and plasma levels of TF (**c**) and serum levels of fructosamine and plasma levels of TF (**d**). DM, type 2 diabetes

Participants in the type 2 diabetes group compared with the control group had 59% higher plasma concentrations of FVIIa-AT (median [IQR] 89 [79–112] vs 56 [48–71] pmol/l, *p* < 0.0001) but not TF (median [IQR] 1.38 [1.26–1.53] vs 1.29 [1.17–1.44] pmol/l, *p* = 0.07) or F1+2 (median [IQR] 196 [146–238] vs 182 [172–192] pmol/l, *p* = 0.42). However, diabetic participants with HbA_1c_ <48 mmol/mol (<6.5%), compared with those with HbA_1c_ ≥48 mmol/mol (≥6.5%), had slightly lower plasma TF and FVIIa-AT concentrations (Fig. 5a). No difference for F1+2 was observed (median [IQR] 188 [97–255] vs 190 [148–217] pmol/l, *p* = 0.66).

Only in the diabetic participants we found a positive association between plasma FVIIa-AT and serum fructosamine levels (Fig. 5b), while plasma TF correlated positively with both HbA_1c_ (Fig. 5c) and fructosamine (Fig. 5d). No associations between F1+2 and HbA_1c_ or fructosamine levels were found. Similarly, no correlations of plasma TF, FVIIa-AT or F1+2 with echocardiographic variables in participants with or without type 2 diabetes were noted (data not shown).

### In vitro studies

#### Expression of NF-κB and BMP-2 in VICs cultures

**Fig. 6:**
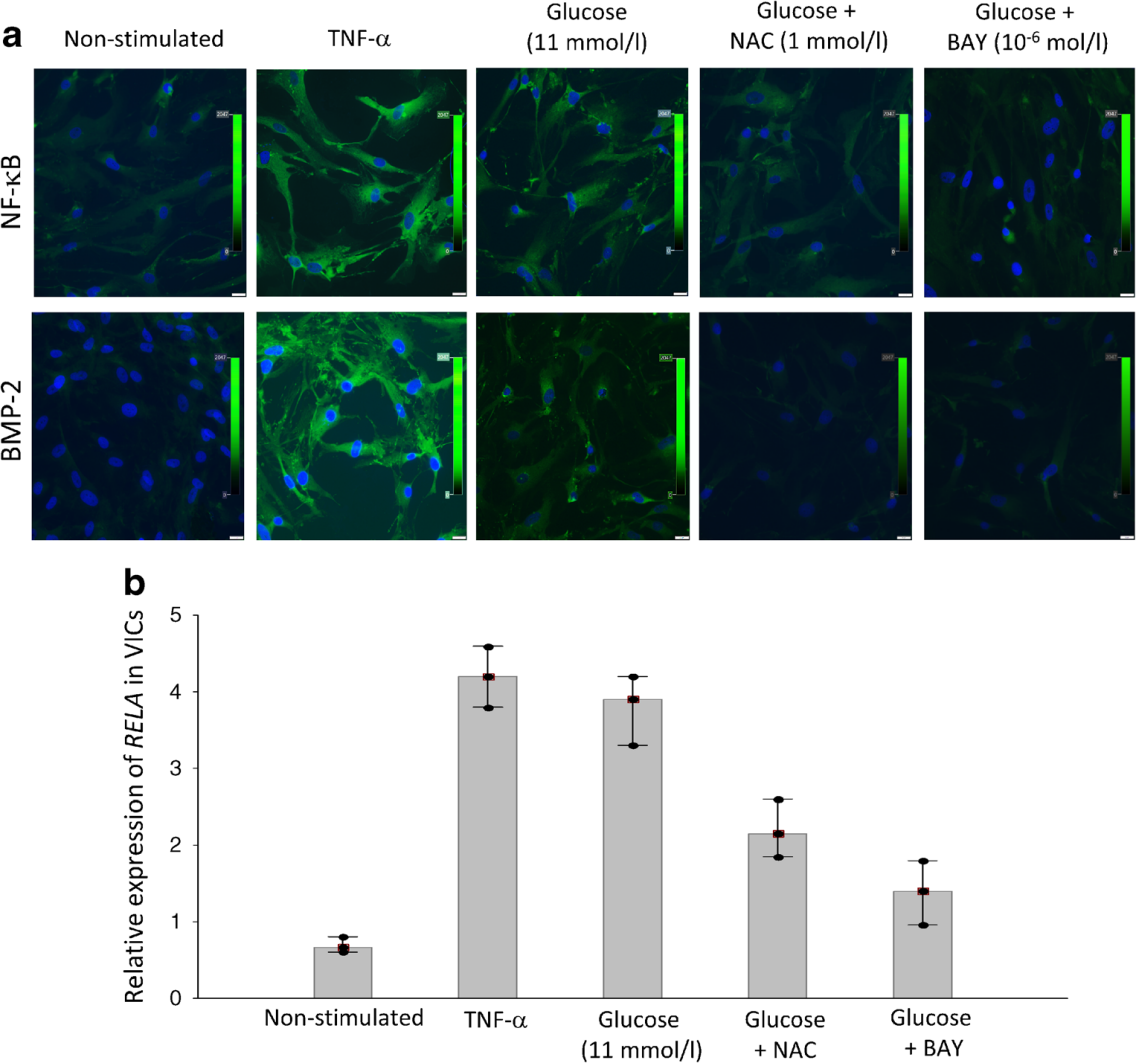
The influence of glucose (11 mmol/l) and specific inhibitors of ROS (NAC) and transcription pathway NF-κB (BAY 11-7082 [BAY]) on the expression of NF-κB and BMP-2 in VICs isolated from aortic stenotic valves obtained during surgery. (**a**) Representative microphotographs of immunostaining in VIC cultures. Scale bar, 20 μm. (**b**) Relative expression of *RELA* in VIC cultures after stimulation. Data are presented as mean±SD

VICs activated with TNF-α showed upregulated expression of NF-κB (+75 ± 10%, *p* < 0.001) accompanied by higher expression of BMP-2 (+80 ± 12%, *p* < 0.001) when compared with unstimulated cells (Fig. 6a). A comparable effect was observed after incubation of VICs at high glucose concentration (+56 ± 10% for NF-κB and +52 ± 9% for BMP-2, both *p* < 0.001). The expression of NF-κB was downregulated in VICs incubated with glucose plus ROS inhibitor (−29 ± 7%, *p* < 0.01) or NF-κB inhibitor (−31 ± 7%, *p* < 0.01) (Fig. 6a). A similar effect was observed for BMP-2 expression after treatment of VICs with glucose plus ROS inhibitor (−31 ± 8%, *p* < 0.01) or NF-κB inhibitor (−33 ± 8%, *p* < 0.01) (Fig. 6a).

#### Relative expression of NF-κB mRNA in VICs

Stimulation of VICs by TNF-α resulted in a 7.8-fold increase in *RELA* expression compared with non-stimulated VICs cultures (Fig. 6b). VICs treated with glucose showed a 6.9-fold increase in *RELA* expression compared with no treatment, while pre-incubation of VICs with glucose in combination with ROS or NF-κB inhibitors suppressed the *RELA* expression by 1.8-fold and 3.2-fold compared with VICs treated with glucose alone (Fig. 6b).

## Discussion

This study is the first to demonstrate that individuals with severe isolated AS and concomitant type 2 diabetes, compared with no concomitant diabetes, exhibit enhanced valvular expression of NF-κB in association with increased expression of valvular FII, FXa and BMP-2. In diabetic individuals, valvular expression of NF-κB correlated with PG_max_, AVA and biomarkers of long-term glycaemic control, namely HbA_1c_ and fructosamine*.* Poorly controlled type 2 diabetes was associated with the highest in loco expression of inflammatory and calcification markers, as well as higher concentrations of plasma coagulation factors, such as TF and FVIIa-AT. Moreover, in vitro experiments conducted on VICs isolated from stenotic aortic valves confirmed that high concentrations of glucose generate inflammation through NF-κB-mediated signalling, leading to subsequent cellular calcification. We also showed that inhibition of either ROS or NF-κB prevents VICs calcification. These data are in line with our previous report showing that AS patients with poorly controlled type 2 diabetes are characterised by higher transvalvular pressure gradients and higher valvular accumulation of AGEs associated with AS severity and serum levels of HbA_1c_ and fructosamine [18].

Previous reports on the association between diabetes and the incidence of AS progression are inconsistent [27–31]. Aronow et al [27] and Kamalesh et al [28] showed a positive association between diabetes and AS progression in individuals with mild and moderate AS, respectively, but no such data are available for severe AS. Katz et al [29] found that both diabetes and the metabolic syndrome were independently associated with an increased prevalence of valvular calcification. Finally, an increased risk of AS development in individuals with type 2 diabetes was shown by Larsson et al [30] in a cohort study comprised of more than 70,000 participants. Testuz et al [31] found no association between AS progression (in individuals with at least mild asymptomatic AS) and diabetes or the metabolic syndrome. However, in their study, only short-term glucose control (reflected by fasting glucose levels) was assessed. Arguably, as demonstrated by our previous research [18], long-term glycaemic control may be of key importance. The present data confirmed that only HbA_1c_ and fructosamine were associated with valvular inflammation and calcification, while glucose levels showed only a very weak association. Importantly, the highest in loco expression of both NF-κB and BMP-2 was seen in individuals with poorly controlled diabetes. This data supports the hypothesis that maintaining long-term glycaemic variables within normal values in individuals with type 2 diabetes who have mild-to-moderate AS may slow the rate of AS progression. However, further studies are warranted to elucidate this issue.

Taken together, we propose the following mechanism underlying the influence of type 2 diabetes on AS progression: hyperglycaemia leads to enhanced accumulation of AGEs/receptor for AGEs (RAGE) and, as a consequence, enhanced production of ROS within valves [18]. Further, ROS escalate valvular inflammation via aggravated macrophage activation and NF-κB pathway expression with upregulated expression of BMP-2-4, osteopontin, osteocalcin, Smad1/5/8, and Runt-related transcription factor 2 (Runx-2), resulting in increased calcium deposition [32]. The findings by Vadana et al [32] are in line with our hypothesis. They showed that high glucose concentration (25 mmol/l) resulted in remodelling of VICs, defined as increased production of matrix metalloproteinases and extracellular matrix proteins, and increased expression of proinflammatory cytokines [32, 33], cell adhesion molecules and integrins [33]. Since inhibition of the NF-κB pathway not only decreased NF-κB expression at the protein and mRNA level but also decreased BMP-2 expression, the present study extended observations by Vadana et al [32] and showed that glucose-driven VIC activation is mediated via the NF-κB pathway and might be responsible for faster valve calcification and dysfunction.

While these findings bear the limitations inherent to flow [3] and observer-dependent echocardiographic measurements, one can speculate that they reflect a more pronounced expression of NF-κB in individuals with a heavier calcific burden on the aortic valve. Optimally, our findings should be verified by a flow-independent method of calcification assessment, like computed tomography (CT)-based calcium scoring [34].

### Coagulation

We are the first to show that individuals with type 2 diabetes and AS have significantly higher valvular expression levels of FII and FXa. Moreover, poorly controlled diabetes was associated with the highest plasma TF and FVIIa-AT concentrations.

It has been shown that increased accumulation of AGEs/RAGEs is able to increase TF expression [35], platelet aggregation [36, 37] and fibrin stabilisation, and reduce the sensitivity of fibrin to degradation by plasmin [37, 38]. The current data suggests that poorly controlled diabetes is associated with a systemic prothrombotic state that can influence AS severity. However, we did not find enhanced thrombin generation in the participants with type 2 diabetes. As the associations between type 2 diabetes and its complications are rather longitudinal, one might hypothesise that prolonged exposure to hyperglycaemia predisposes to a more extensive calcific burden. Apparently, a diabetic individual may have a more calcified valve compared with a non-diabetic individual at the time of symptom presentation and surgical intervention. It remains to be established how diabetes biologically affects AS at the earlier stages of the disease. This is technically more difficult, as the surgical removal of the diseased valve is warranted at the very late stage of disease progression in isolated AS.

### Study limitations

This study has several limitations. Any significant atherosclerosis was used as an exclusion criterion, although the role of atherosclerosis cannot be completely omitted. First, the number of participants in the subgroups with well and poorly controlled diabetes was small. However, this is a unique cohort of a relatively high number of individuals with poorly controlled type 2 diabetes concomitant to AS. Second, we did not assess all haemostasis-related proteins, such as von Willebrand factor, which was shown by Ljungberg et al [39] to be implicated in AS development and thus may influence valvular inflammation. Valvular expression of particular factors was determined semi-quantitatively and therefore these estimations may be less precise. However, microscopic analyses were performed by two independent experienced investigators. Moreover, the presented analysis cannot determine whether type 2 diabetes enhances the expression of the investigated proteins in valvular cell populations other than VICs, as this was beyond the scope of this study. Third, modification of VIC culture conditions, such as glucose concentration or different incubation times, might be considered in order to investigate the longitudinal action of glucose on VICs. It would also be of interest to conduct in vitro studies using co-culture of VICs and macrophages in order to examine the crosstalk between these two cell populations co-existing within stenotic aortic valves. In our opinion, the effect of glucose in co-culture could be even more intense. Finally, this study was performed in individuals with isolated severe AS and our results cannot be extrapolated to individuals with mild or moderate AS. Moreover, AS severity was measured as transvalvular gradients and AVA but not as a peak velocity, which is currently recommended for assessing AS severity [23].

### Conclusions

The current study showed that type 2 diabetes enhances valvular expression of NF-κB and activation of coagulation within aortic stenotic valves and in circulating blood. Enhanced NF-κB expression was associated with AVA and PG_max_. The level of valvular NF-κB expression was associated with HbA_1c_ and fructosamine levels, strongly supporting the concept that strict long-term glycaemic control is needed in AS patients with concomitant type 2 diabetes. Whether maintaining these variables within the normal range might slow the rate of AS progression at earlier stages in the setting of diabetes remains to be established.

## Supplementary information

ESM (PDF 325 kb)

## Abbreviations

AS: Aortic stenosis
AVA: Aortic valve area
BMP: Bone morphogenetic protein
CRP: C-reactive protein
F1+2: Prothrombin fragments 1+2
FII: Prothrombin
FVIIa-AT: Active factor VIIa–antithrombin complex
FXa: Active factor X
NAC: *N*-Acetyl-l-cysteine
PG_max_: Maximal transvalvular pressure gradient
PG_mean_: Mean transvalvular pressure gradient
RAGE: Receptor for AGEs
ROS: Reactive oxygen species
TF: Tissue factor
VICs: Valve interstitial cells
