# Supplementary material for: Diabetes concomitant to aortic stenosis is associated with increased expression of NF-κB and more pronounced valve calcification
Source: Diabetologia. 2021 Sep 7;64(11):2562–74. doi: 10.1007/s00125-021-05545-w (PMC8494674; doi:10.1007/s00125-021-05545-w)
Supplement: Supplementary file 1 — (PDF 325 kb) [file 125_2021_5545_MOESM1_ESM.pdf]

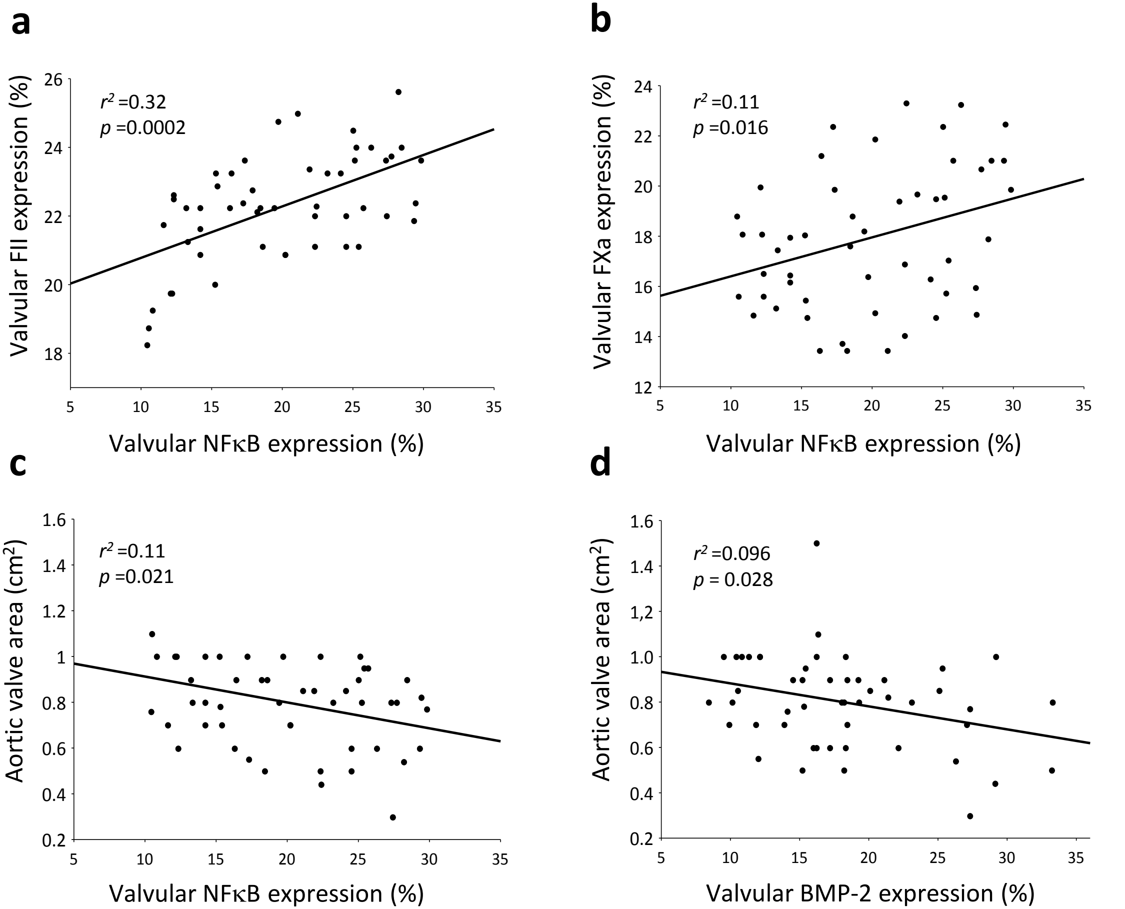


ESM Figure 1. Associations between valvular expression of NFκB and (a) prothrombin (FII), (b) active FX (FXa), and (c) AS severity measured as aortic valve area as well as association between expression of (d) valvular BMP-2 and AS severity in participants with AS but without diabetes.
